# Design, Property, and Interfacial Bonding Mechanism of a Novel Gangue-Cemented High-Porosity Backfill Material

**DOI:** 10.3390/ma19173666

**Published:** 2026-08-28

**Authors:** Qiang Guo, Meng Li, Feng Ju, Zhangjie Yin, Zhangyu Li, Zhibo Cui

**Affiliations:** 1 Sate Key Laboratory for Fine Exploration and Intelligent Development of Coal Resources, School of Mines, China University of Mining and Technology, Xuzhou 221116, China; stu-gq@cumt.edu.cn (Q.G.); ts24020066a31@cumt.edu.cn (Z.Y.); ts25020158p31@cumt.edu.cn (Z.L.); ts25020012a31@cumt.edu.cn (Z.C.); 2 State Key Laboratory of Intelligent Construction and Healthy Operation & Maintenance of Deep Underground Engineering, China University of Mining and Technology, Xuzhou 221116, China; juf1983@163.com

**Keywords:** coal gangue, fast-setting cementitious material, high porosity, backfill material, interfacial transition zone

## Abstract

In traditional gangue backfilling mining, the use of gangue as backfill material often leads to issues such as supply–demand imbalance, low crushing efficiency, and insufficient early strength. To address these challenges, a novel gangue-cemented high-porosity backfill material (GCBM) was developed, using coal gangue (CG, 15–20 mm) as aggregates bonded with an alkali-activated modified coal gangue powder and slag-based fast-setting cementitious material (FCM). The effects of alkali equivalent on the workability and mechanical properties of FCM were investigated to determine the optimal composition. The influence of FCM proportion on the porosity and strength of GCBM was analyzed, and a strength–porosity correlation model was established. The microstructure and bonding mechanism of the interfacial transition zone (ITZ) between FCM-CG aggregates were revealed through multiple microscopic techniques. Results indicate an optimal alkali equivalent of FCM is 6%. The FCM proportion is positively correlated with the compressive and flexural strengths of GCBM, with a critical proportion of 24% at which flexural strength increased by 172% compared with 22%, reflecting enhanced interfacial bonding. Microscopic observations further show that increasing the FCM proportion optimizes the ITZ structure and promotes secondary hydration products, thereby improving interfacial bonding density and the overall mechanical performance of the backfill material.

## 1. Introduction

**Figure 1:**
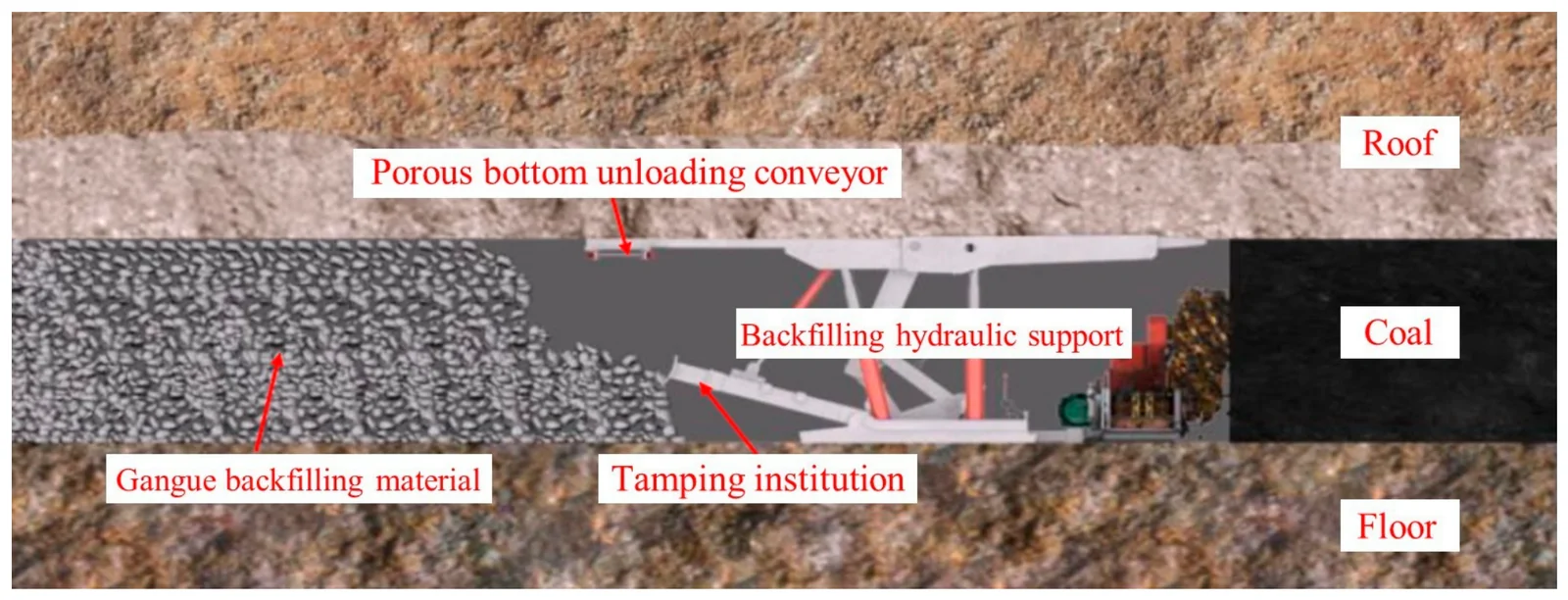
Underground in situ gangue backfilling and mining technology.

The accumulation of solid wastes, particularly coal gangue (CG), from coal mining and utilization poses a significant constraint to the mining industry’s productivity and ecological sustainability [1,2,3]. Statistics show that the output of CG generally accounts for 10% to 25% of that of raw coal in China, and the cumulative volume of stockpiled CG exceeds 7 billion tons [4,5,6]. The traditional stockpiling of CG occupies lots of land resources and may cause environmental pollution [7,8]. In comparison, the in situ backfilling of CG in underground mines can directly use crushed solid waste CG in the underground mined-out area, as illustrated in Figure 1. This method has become one of the key approaches for CG disposal and utilization in mining areas. It can realize multiple objectives, including deformation control of strata and surface, green underground disposal of CG, and ecological protection of the mining area [9,10,11]. However, gangue backfilling mining currently faces two problems [12,13,14]: on the one hand, the supply and demand of backfill materials are imbalanced, with the demand for gangue-based backfill materials being about 1.3~1.5 times the extracted volume of coal, leading to a shortage of these materials for large-scale backfilling mining. On the other hand, the CG crushing is inefficient: to meet the requirement of particle sizes needed for backfilling, the CG generally needs to undergo multi-stage crushing, which is complex and costly, limiting the efficiency of backfilling mining.

To address the aforementioned problems, a novel gangue-cemented high-porosity backfill material (GCBM) was proposed. CG particles of 15~20 mm were taken as the aggregates, which were cemented by fast-setting cementitious material (FCM), thus forming a high-porosity filling body. GCBM shows the following advantages: on the one hand, it adopts large CG particles as the aggregates, apparently simplifying the crushing stage, reducing treatment cost, and overcoming the complexity and low efficiency of traditional multi-stage crushing. On the other hand, the high-porosity structure formed by cementation of large CG particles significantly decreases the consumption of CG per unit volume of filling body while ensuring the load-bearing properties and strata-control ability, thus alleviating the imbalance between the supply and demand of backfill materials.

The mechanical properties of GCBM, as a key load-bearing medium during backfilling mining of coal mines, can directly determine the stability of the stope roof and the controlling effect on the displacement of overlying strata. Existing research mainly focuses on the compression and load-bearing properties of granular CG [15,16,17,18]. For example, Le et al. [15] conducted compression tests to assess the influence of different particle size distributions (PSDs) on the compressive behaviors of granular CG and determined the appropriate PSD envelope; Li et al. [17] modified granular CG by adding fly ash and studied the mechanical strength and deformation of the CG–fly ash mixture with different proportions under load; however, such studies mainly focus on unconsolidated or weakly consolidated granular materials, while the correlation between the porosity and macroscopic mechanical property of novel backfill materials, including GCBM with cemented and high-porosity structures, remains unclear. This limits the material design and engineering application.

Moreover, the performance of GCBM is intrinsically linked to the properties of FCM, which binds the particles and governs the resulting pore structure and mechanical strength. Consequently, the selection of FCM is critical. Conventional Portland cement, despite its widespread use, is often avoided due to its high carbon footprint and energy-intensive production, which present considerable economic and environmental drawbacks [19,20]. To this end, some researchers prepared cementing components using solid wastes such as CG powder, while it generally entails activating treatment due to the low natural activity [21,22,23]. Moussadik et al. [22] found that the combination of mechanical grinding and thermal activation can significantly increase the content of amorphous phases in the CG powder, and the prepared cementing material exhibits a significantly improved compressive strength. However, this method is characterized by slow setting, with the initial setting time being as long as 120~180 min. Consequently, the early strength of filling bodies is generally low, failing to meet the technological requirement for rapid roof contact of the backfill material in underground mines. In comparison, alkali-activated cementing materials provide a new approach for meeting the above requirement by virtue of their advantages, including high early strength, high hydration activity, and controllable binding [24,25,26]; however, their net environmental benefit is context-dependent, as activator production also entails emissions, and it is maximized when integrated with in situ solid-waste utilization. Sasaki et al. [25] found that using NaOH-Na_2_SiO_3_ composite alkali activator can shorten the initial and final setting times of fly ash–blast furnace slag composite slurry to 25 min and 55 min, separately. The three-dimensional (3D) compressive strength reaches 72% of the 28 d strength, showing excellent early binding. However, previous research mainly explores optimization of the properties of alkali-activated cementing materials themselves, and there is a lack of research into their suitability as binders of CG particles and the interfacial bonding mechanism of FCM and CG aggregates: the mechanism underpinning such changes remains to be clarified.

On this basis, a skeleton structure was constructed, taking CG of 15~20 mm as the aggregates, and FCM was prepared using alkali-activated modified coal gangue (MCG) powder and slag powder. Then, the macroscopic–microscopic performance and enhancement mechanism of GCBM were systematically explored. The mechanism underlying the changes in the workability and mechanical property of FCM with the alkali equivalents was studied, and the optimal FCM proportion was determined. The influences of the FCM proportion on the porosity and mechanical properties of GCBM were discussed to further establish the correlation between the porosity and mechanical properties. Then, multiple microscopic characterization methods were adopted to characterize microstructures of the interfacial transition zone (ITZ) between FCM and CG aggregates and expound the mechanism of action of the interfacial effect on the macroscopic properties of GCBM specimens.

## 2. Materials and Methods

### 2.1. Raw Materials

**Figure 2:**
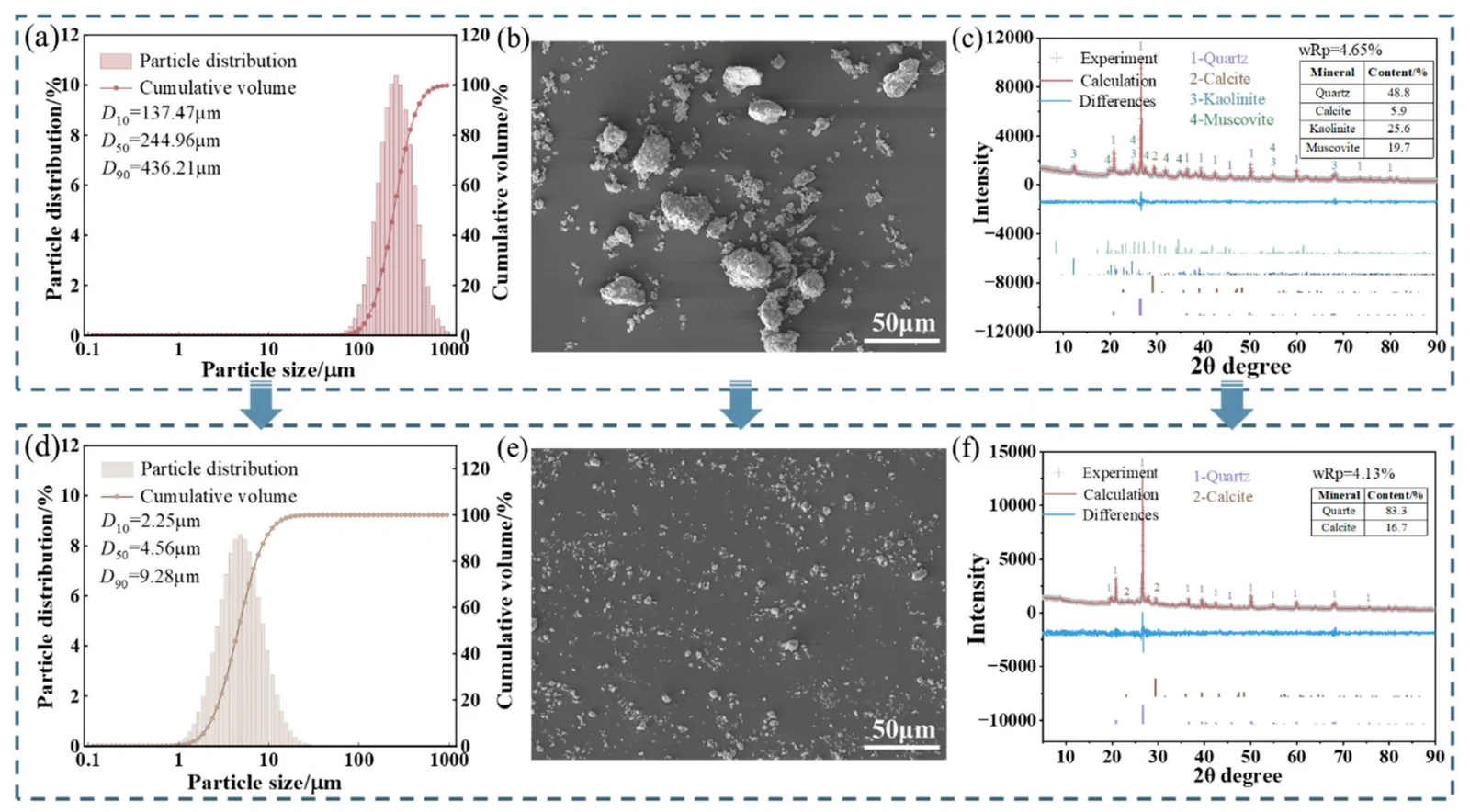
Comparative characterization of unmodified and modified CG: (**a**,**d**) PSD; (**b**,**e**) SEM; and (**c**,**f**) XRD.

CG and blast furnace slag (S) were selected as the raw materials for testing. Therein, the CG was taken from Jinjitan Coal Mine (Yulin, China), and the S95-grade S was obtained from Jiangsu RongDa New Material Co., Ltd. (Nantong, China). The raw CG was first crushed using a jaw crusher to <5 mm, then ball-milled at 450 rpm for 60 min with a ball-to-powder ratio of 5:1. The powder was calcined at 650 °C (heating rate 10 °C/min) for 2 h in a muffle furnace, followed by natural cooling inside the furnace, and finally sieved through a 200-mesh screen to obtain the MCG powder [23]. The chemical compositions of the CG before and after modification are listed in Table 1. The loss on ignition of MCG is shown to reduce significantly, while the contents of SiO_2_, Al_2_O_3_, and CaO increase. The physico-chemical characteristics were further studied using a PSD analyzer (PSD, Jinan, China), scanning electron microscopy (ZEISS, Oberkochen, Germany), and XRD (Rigaku SmartLab SE, Hefei, China) (Figure 2). PSD results show that the particle size of MCG decreases and *D*_50_ is 4.56 μm, which is one to two orders of magnitude smaller than that of CG. SEM observation confirms the occurrence of particle refinement and the increased generation of flocculent structures on the edge of particles. According to the XRD results, the peaks ascribed to kaolinite and muscovite disappear after modification, while the relative amount of quartz increases. The raw CG mainly contains quartz and kaolinite. After modification, the kaolinite and muscovite peaks disappear, and the MCG is dominated by quartz, which, together with the amorphous phases, provides active SiO_2_ and Al_2_O_3_ for the subsequent alkali-activated reaction.

The alkali activator used in the experiment was prepared using liquid sodium silicate and solid sodium hydroxide. Therein, the modulus of sodium silicate was 3.3, containing 26.98% of SiO_2_ and 8.53% of Na_2_O, with a density of 1.366 g/mL; the purity of sodium hydroxide was 96%.

### 2.2. Specimen Preparation

**Figure 3:**
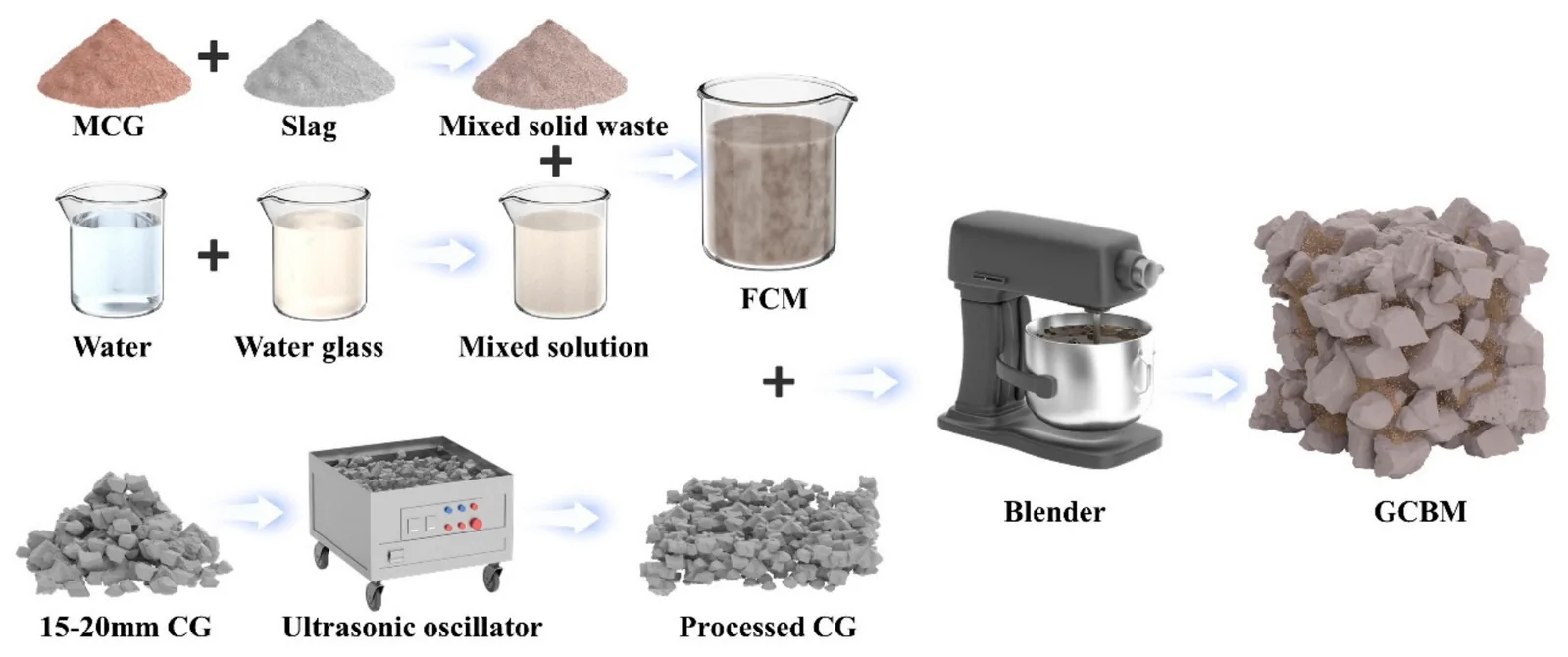
GCBM sample preparation process.

The sample preparation process is shown in Figure 3, and it included two steps:

(1) Preparation of FCM: The MCG and S were mixed at a mass ratio of 1:1. Then, sodium silicate with the modulus of 1.4 was blended with water and stirred for 20 min, followed by pouring in the mixed MCG and S. A blender was used for slow stirring for 20 s and rapid stirring for 20 s, thus obtaining FCM. The water–binder ratio of FCM was set to 0.5, and the alkali equivalents were 0%, 1%, 2%, 4%, 6%, and 8% (specimens labeled FCM0% to FCM8%).
(2) Preparation of GCBM: The 15–20 mm CG aggregates used in this study have an apparent density of approximately 2.56 g/cm^3^, a bulk density of approximately 1360 kg/m^3^, a water absorption rate of approximately 5.3%, and a crushing index of approximately 12%. CG particles were placed in an ultrasonic oscillator to be oscillated for 30 min under an applied power of 200 W. After draining off the water, the CG particles were oven-dried at 60 °C for 12 h, which can reduce the dust on and improve the cleanliness of CG surfaces; thereafter, the prepared FCM was poured over CG particles, and the mixture was stirred sufficiently so that CG particles became coated with FCM. The mixture was then poured into molds lubricated with grease and oscillated for 5 s, thus obtaining the GCBM. The FCM proportions (proportions of FCM in GCBM) were set to 20%, 22%, 24%, 26%, and 28% (specimens labeled GCBM1 to GCBM5), as shown in Table 2. After demolding, all specimens were cured in a standard curing room at 20 °C ± 2 °C, with a relative humidity of ≥ 95% until the specified testing age.

### 2.3. Test Methods

#### 2.3.1. Rheological Test

**Figure 4:**
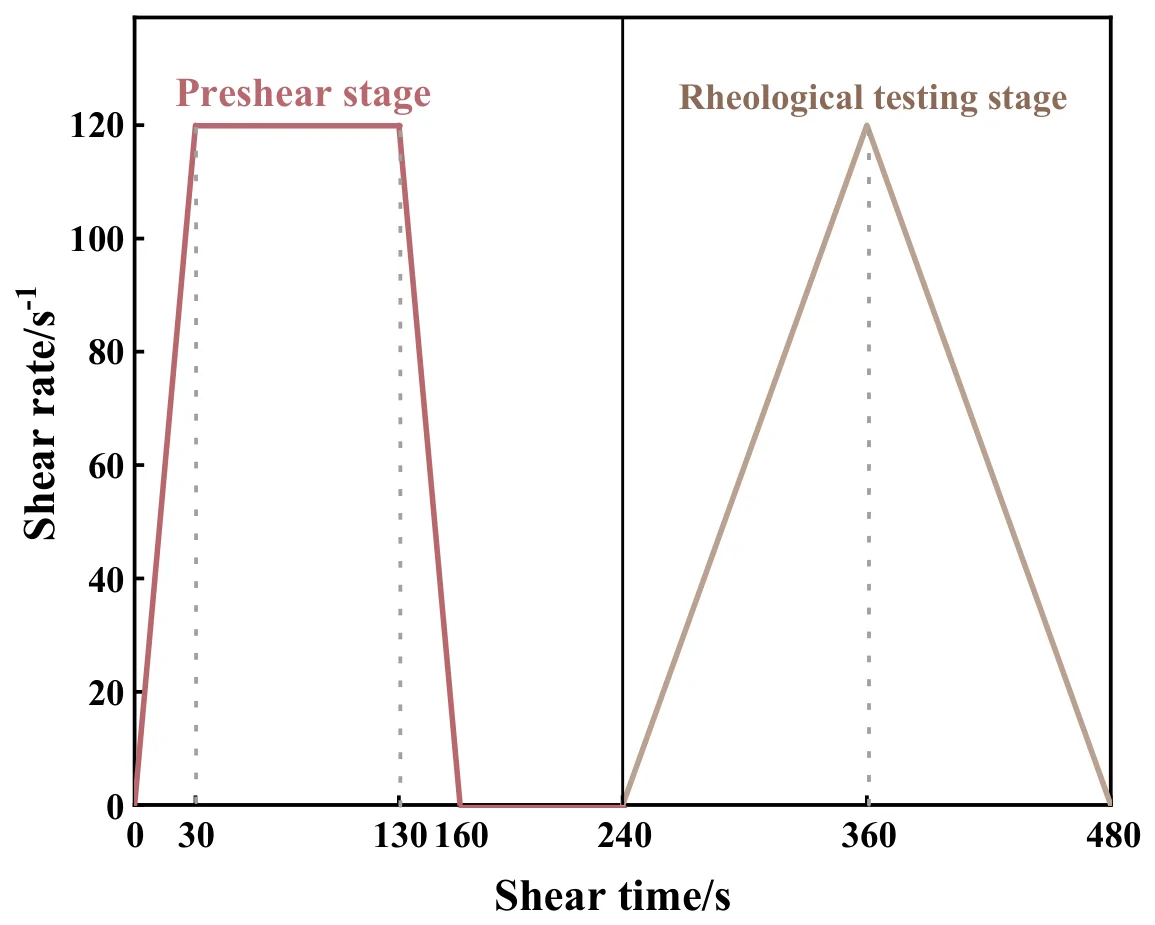
The rheological testing procedure.

The FCM rheology was characterized per the method of Gu et al. [27] and illustrated in Figure 4. The test involved two consecutive stages: (1) A 240 s preshear stage for slurry homogenization, executed by linearly ramping the shear rate to 120 s^−1^ (0–30 s), maintaining it (30–130 s), linearly reducing it to zero (130–160 s), and allowing an 80 s rest (160–240 s); (2) A 240 s data acquisition stage to record the flow hysteresis, where the shear rate followed a predefined ramp: 0 to 120 s^−1^ in 120 s, immediately followed by a decrease to 0 s^−1^ in 90 s. A total of 120 data points were sampled during this stage.

#### 2.3.2. Porosity Test

The porosity of GCBM specimens can be calculated using Equation (1):

(1) $$P=(1-\frac{m_{1}}{\rho_{1}V_{0}}-\frac{m_{2}-m_{1}}{\rho_{2}V_{0}})\times 100\%$$

where *P* is the porosity of GCBM specimens (%); *m*_1_ is the total mass of CG in the mold (g); *ρ*_1_ denotes the CG density (g/cm^3^); *V*_0_ denotes the volume of the mold (cm^3^); *m*_2_ is the total mass of GCBM (g); *ρ*_2_ is the density of the FCM (g/cm^3^).

#### 2.3.3. Setting Time Test

The initial and final setting times of FCM pastes were determined using a Vicat apparatus manufactured by Xuzhou Ailina Instrument & Meter Technology Service Co., Ltd. (Xuzhou, China). The FCM paste was prepared according to the mix proportions given in Section 2.2 and then placed in the Vicat mold at 20 ± 2 °C and RH ≥ 60%. The initial setting time was recorded when the Vicat needle penetration depth reached 36 ± 1 mm from the bottom of the mold, while the final setting time was recorded when the needle penetrated no more than 0.5 mm into the paste surface. Each test was repeated three times, and the average values are reported.

#### 2.3.4. Mechanical Testing

The uniaxial compressive strength was evaluated for specimens cured for 3, 7, and 28 d. After surface grinding, the specimens were loaded at a constant displacement rate of 0.5 mm/min using a WDW-600WE universal testing machine manufactured by Jiangsu Bainai Scientific Instruments Co., Ltd. (Xuzhou, China) until failure, with the peak stress being recorded. Each data point represents the average of at least three valid tests, with outliers excluded to ensure reliability. The flexural strength was determined using a three-point bending configuration with a span of 100 mm and a loading rate of 0.5 mm/min. For each mix proportion, at least three specimens were tested, and the average value was recorded.

#### 2.3.5. Analysis of Mineral Compositions

To analyze the mineral composition, the samples were characterized by XRD (Rigaku SmartLab SE, Hefei, China) after a series of pre-treatment steps. This involved immersion in anhydrous ethanol for 24 h with a change of ethanol every 12 h, followed by vacuum drying at 45 °C for 24 h and pulverization to 200 mesh. The XRD analysis was conducted with a scanning range of 5° to 70° at a speed of 2°/min.

#### 2.3.6. Analysis of Micro-Morphologies

Following a 28-day curing period, the GCBM specimens were prepared for microstructural analysis by first immersing them in absolute ethyl alcohol for 24 h to arrest hydration. Subsequently, the samples were vacuum dried at 45 °C for 24 h, impregnated with epoxy resin, and sequentially ground with P240 to P2000 mesh metallographic abrasive paper. A final polish was achieved using a 3.5 μm diamond suspension. The interfacial characteristics of the CG aggregates were then examined via BSE-EDS imaging using a scanning electron microscope (ZEISS, Oberkochen, Germany).

## 3. Experimental Results

### 3.1. Optimization of Alkali Equivalent in FCM

#### 3.1.1. Workability

**Figure 5:**
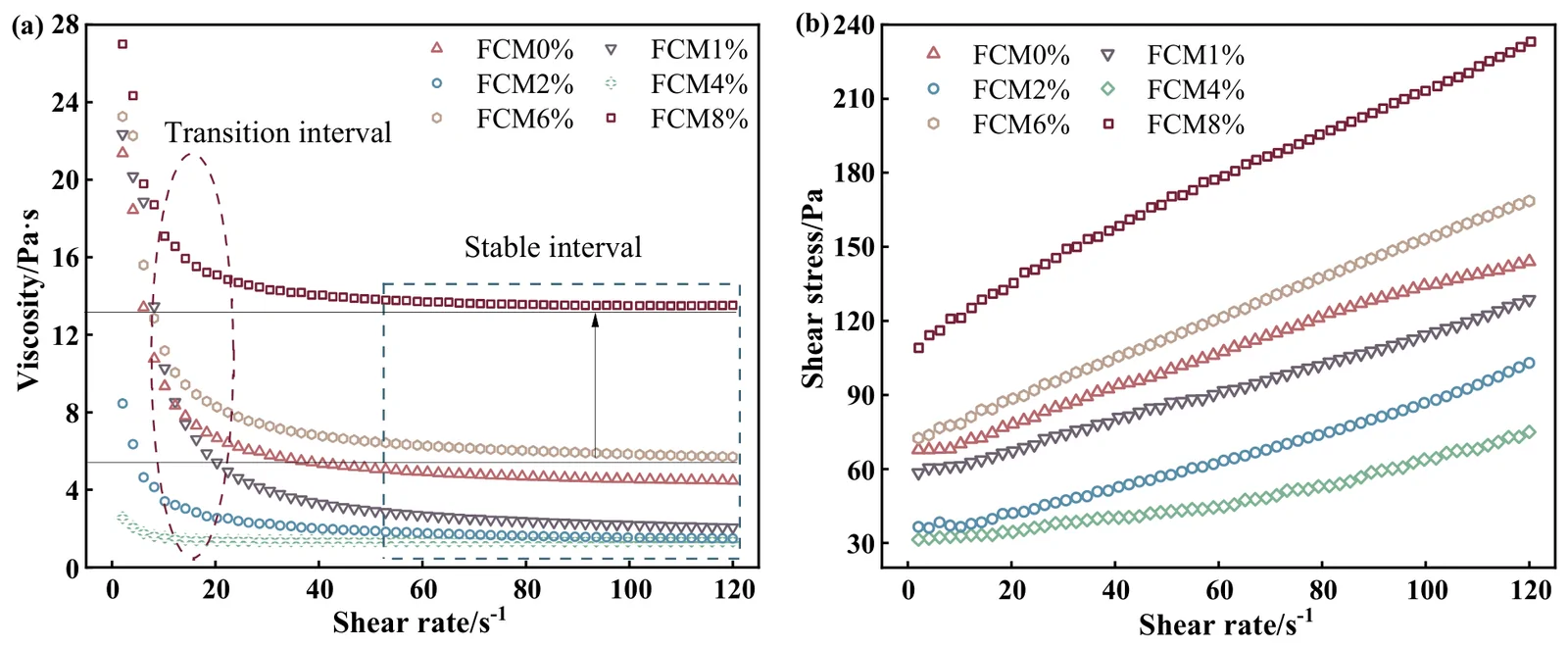
Rheograms of FCM mixtures with different alkali equivalents: (**a**) apparent viscosity and (**b**) shear stress.

The workability of FCM not only directly determines the transportation and construction efficiency of newly mixed FCM but also affects the bonding quality of ITZ in the formation of GCBM, thus affecting the mechanical properties of GCBM. Figure 5 illustrates the rheological curves of FCM under different alkali equivalents. As the shear rate rises, the apparent viscosity of FCM exhibits a sharp drop, a trend that is universal for all tested alkali equivalents (Figure 5a), and then stabilizes (i.e., exhibits shear thinning). The behavior stems from the dynamic evolution of the reticulated floc structure in the slurry under shear: the shear force damages this structure, which is reconnected under the interparticle force. The damage and repair compete and finally reach an equilibrium, as evinced by the stabilization of the apparent viscosity [28]. The apparent viscosity of FCM demonstrates a non-monotonic dependence on alkali equivalent. An initial decrease is observed within the 0% to 4% range. This reduction is attributed to elevated Na^+^ concentration, which diminishes interparticle steric hindrance and enhances the lubricating effect of the liquid phase, thereby improving slurry fluidity and lowering viscosity [29]. However, a further increase in alkali equivalent causes a sharp rise in viscosity. This reversal is due to the high OH^−^ concentration promoting the dissolution of MCG and slag (S), which accelerates hydration and the formation of a gel network that significantly increases resistance to flow [30]. Under an alkali equivalent of 8%, the stabilized apparent viscosity of FCM reaches 13.5 Pa·s, which is 137% higher than that under an alkali equivalent of 6%, while too high a viscosity is not conducive to efficient transportation and workability of the slurry [31,32].

To quantitatively analyze the rheological parameters (yield stress and plastic viscosity), the data in Figure 5b were fitted with the Herschel–Bulkley (H-B) model [33]. The H-B model can be expressed using Equation (2):

*τ* = *τ*_0_ + *ηγ^n^* (2)

The variables in Equation (2) are defined per Gao et al. [33]. The resulting rheological parameters derived from the H-B model are summarized in Table 3. All fitted values for the flow index (*n*) are below unity, confirming the shear thinning behavior of the slurry.

**Figure 6:**
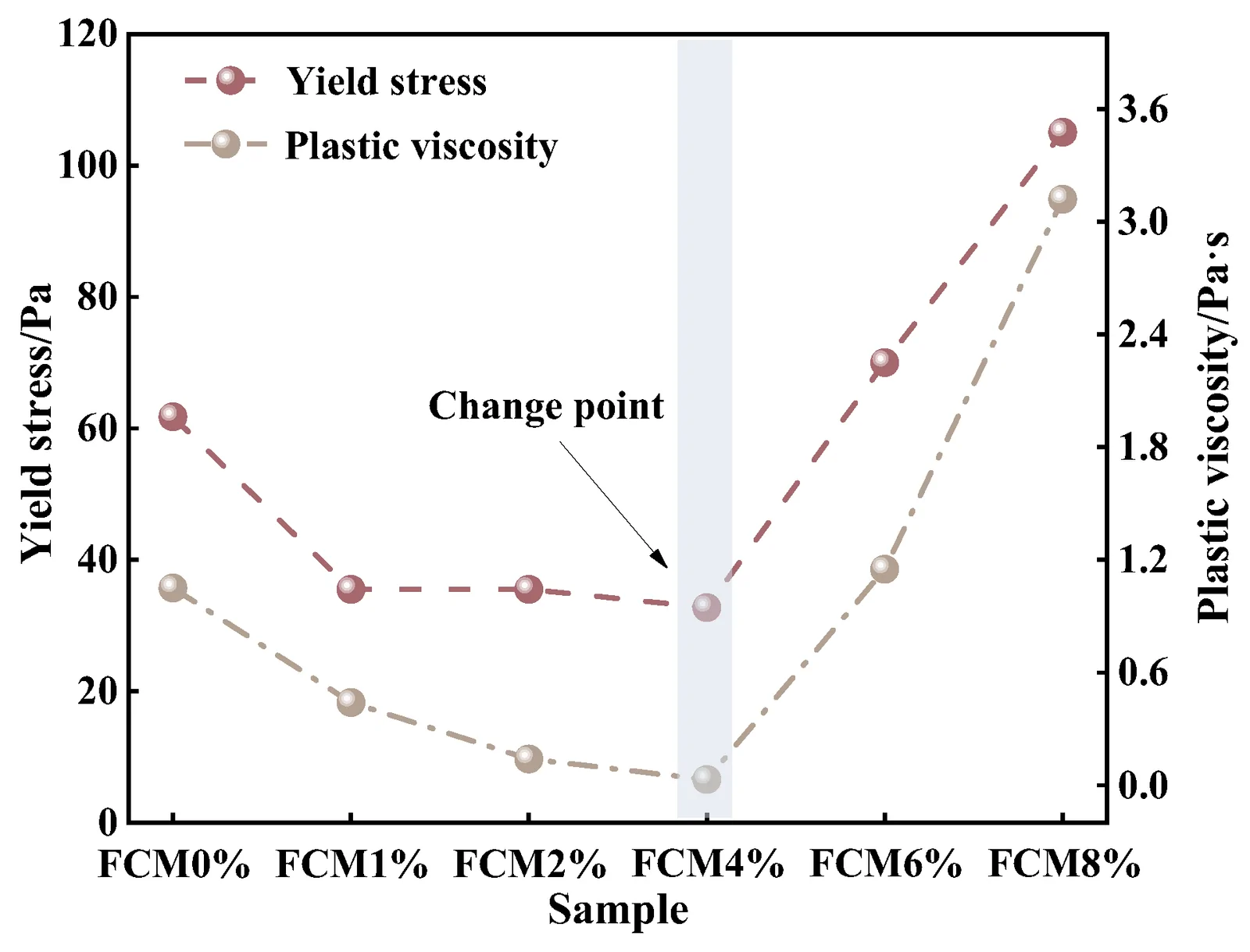
Relationships of the yield stress and plastic viscosity with the alkali equivalent.

The yield stress and plastic viscosity, which define the slurry’s deformation threshold and internal flow resistance, respectively [34,35], collectively govern the rheology of FCM. As shown in Figure 6, both parameters initially decrease, reaching minimum values at a 4% alkali equivalent (yield stress: 32.75 Pa; plastic viscosity: 0.03 Pa·s), before increasing significantly beyond this critical point. This trend is governed by two factors: first, high alkali content accelerates hydration, consuming free water, thinning interparticle water films, and increasing friction [36]; second, the inherent viscosity of sodium silicate in the activator directly elevates the slurry’s overall viscosity [37].

**Figure 7:**
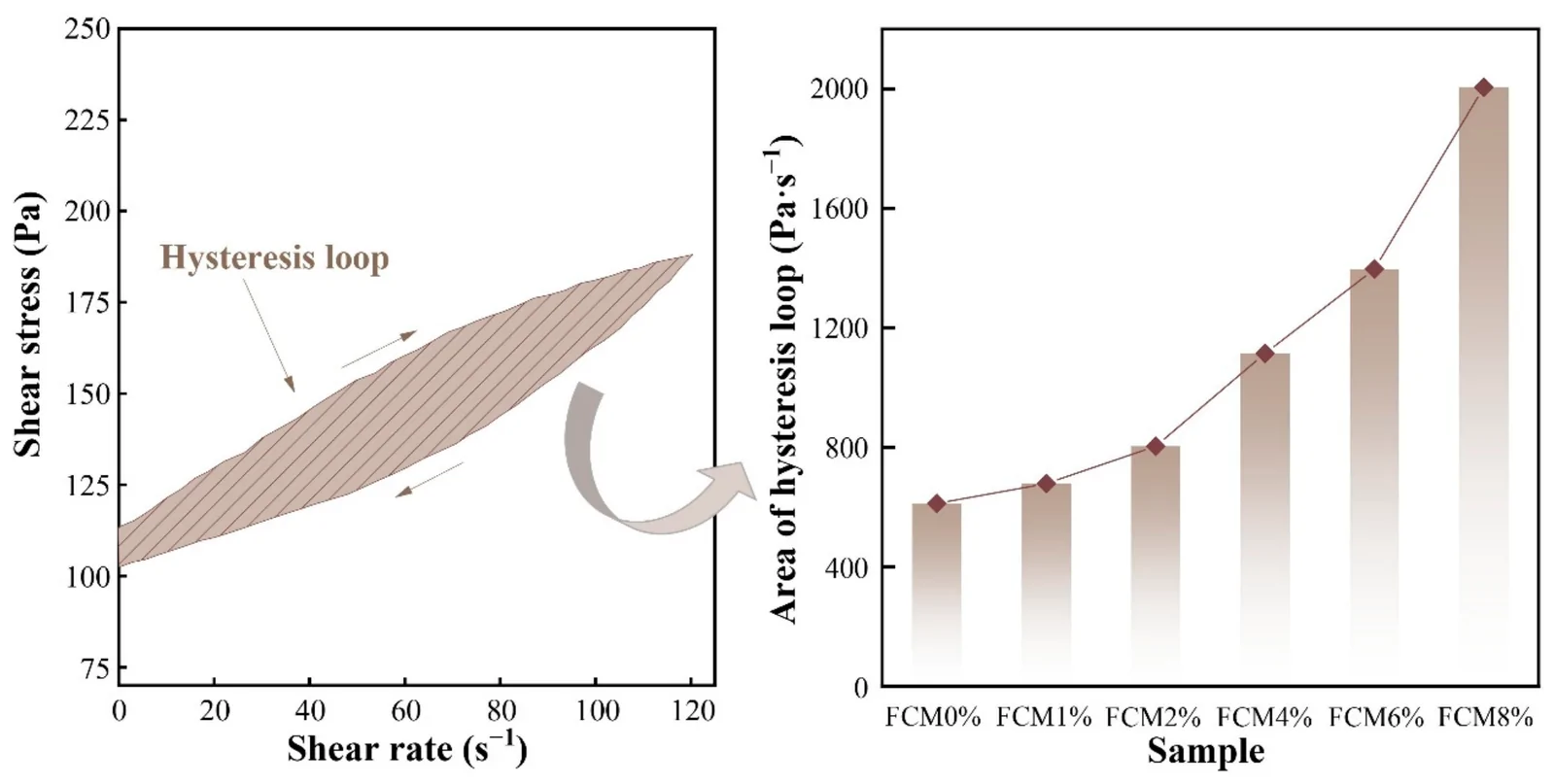
Evolution of the hysteresis loop area with alkali equivalent.

The rheological curves of FCM exhibit a hysteresis loop between the ascending and descending shear rate paths, a characteristic of thixotropy. The area enclosed by this loop, which quantifies the slurry’s thixotropy, reflects the breakdown and recovery of its internal flocculated network [38]. As shown in Figure 7, the hysteresis area expands with increasing alkali equivalent, with a pronounced 43.5% surge from 1397 to 2005 Pa·s^−1^ between 6% and 8%. This abrupt change is attributed to the high OH^−^ concentration, which drastically accelerates the dissolution and polycondensation of active Si-Al components. Consequently, gel networks are rapidly formed and densified in the system and significantly enhance the resistance to structural restoration and thixotropic properties of the slurry [39].

**Figure 8:**
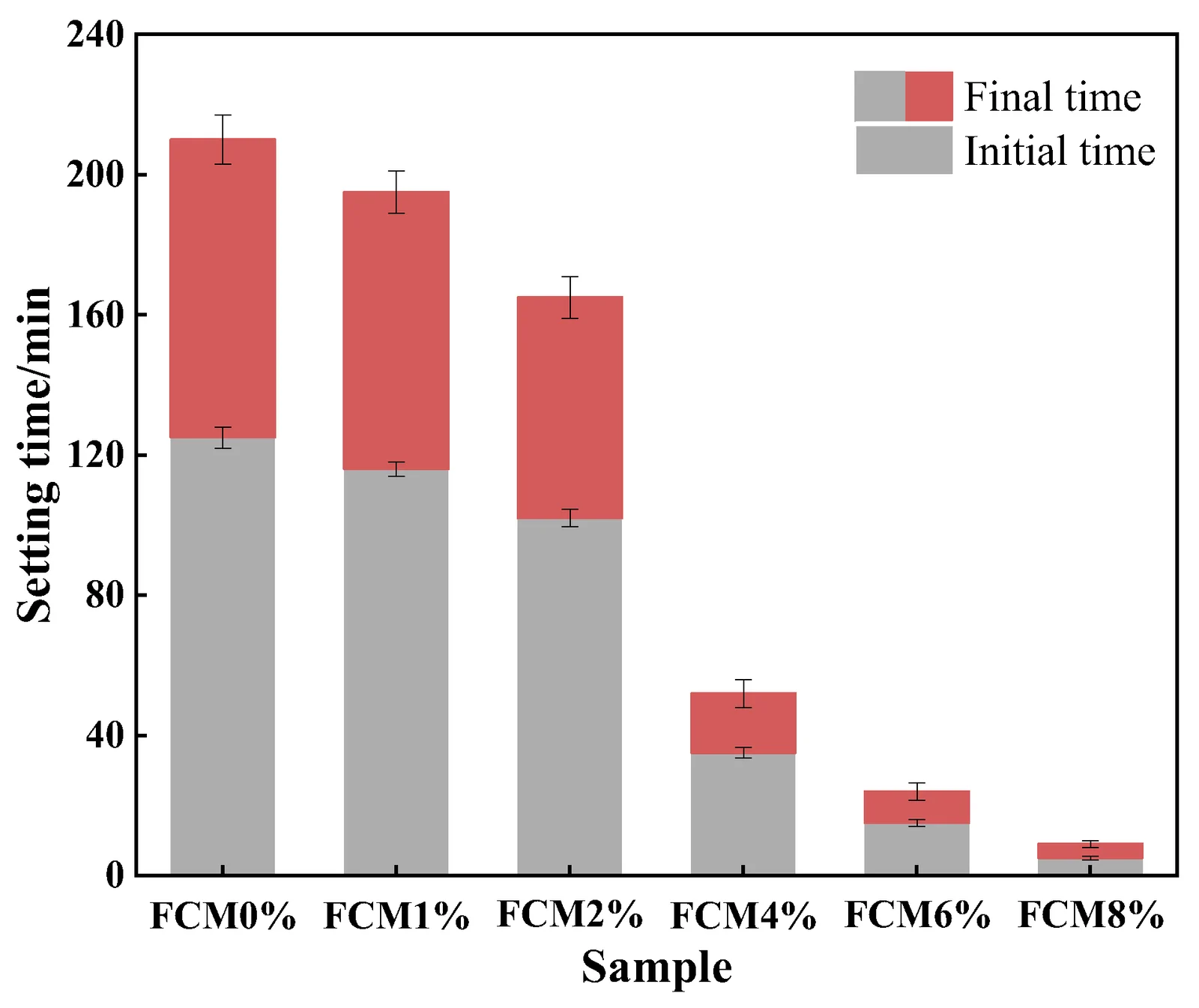
Effect of alkali equivalent on the initial and final setting times of FCM.

The setting times of FCM exert decisive influences on the bonding strength, construction adaptability, and long-term durability. If FCM is set too quickly, the hydration reaction may be insufficiently advanced, thus forming porous, defective structures; if FCM is set too slowly, it delays the development of early strength and weakens the interfacial bonding effect [40]. Precise control of the setting process is therefore critical for the engineering application of FCM. Figure 8 demonstrates a consistent shortening of both initial and final setting times with increasing alkali equivalent. A notable abrupt change occurs between 2% and 4%, where the initial setting time plummeted from 102 min to 35 min and the final setting time from 165 min to 52 min. This transition is triggered once alkalinity surpasses a critical threshold, prompting a shift from “local erosion” to “overall depolymerization” of Si–O and Al–O bonds. This dramatically accelerates the dissolution of MCG and S, facilitating the nucleation and growth of hydration products and thereby curtailing the setting period [41]. At an 8% equivalent, the setting times are drastically reduced to merely 5 min and 9 min, respectively. Such rapid setting compromises the material’s compliance with practical transportation and workability requirements [42].

#### 3.1.2. Mechanical Properties

**Figure 9:**
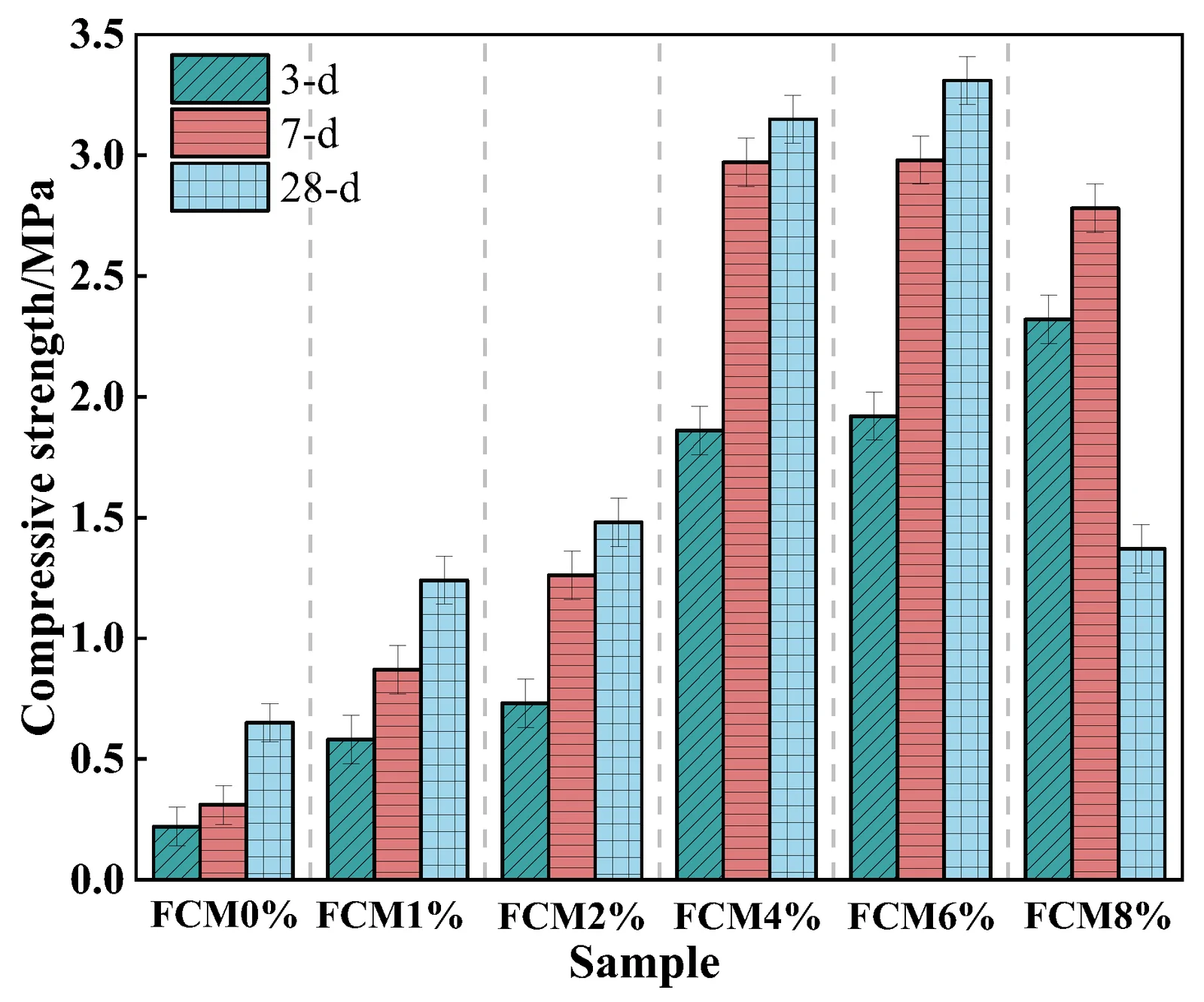
Effect of alkali equivalent on the compressive strength of FCM.

The early-age compressive strength, a critical indicator for the engineering application of FCM, demonstrates a consistent increase in both 3 d and 7 d values as the alkali equivalent rises from 0% to 8% (Figure 9). This trend confirms that a higher alkali content effectively promotes the early-age hydration reaction and strength development. However, although the early strength (3 d and 7 d) under an alkali equivalent of 8% does not greatly differ from that under an alkali equivalent of 6%, the 28 d strength decreases significantly (being 49% lower than the 7 d strength and obviously lower than the group with the alkali equivalent of 6%). The strength decline stems from excessively rapid hydration kinetics caused by high alkalinity. This leads to the formation of a non-uniform gel network and elevated shrinkage stress, which promotes micro-cracking in later stages and consequently degrades the mechanical properties of FCM [43].

Combining with the test results relating to workability in Section 3.1.1, the alkali equivalent was finally determined to be 6%, which was used to prepare FCM. Under this alkali equivalent, FCM shows high plastic viscosity and short initial setting time. It exhibits the excellent early strength and late load-bearing stability while ensuring the favorable transportation property, which meets the comprehensive requirements for the construction and mechanical properties of backfill materials [44,45].

### 3.2. Properties of GCBM

#### 3.2.1. Porosity

**Figure 10:**
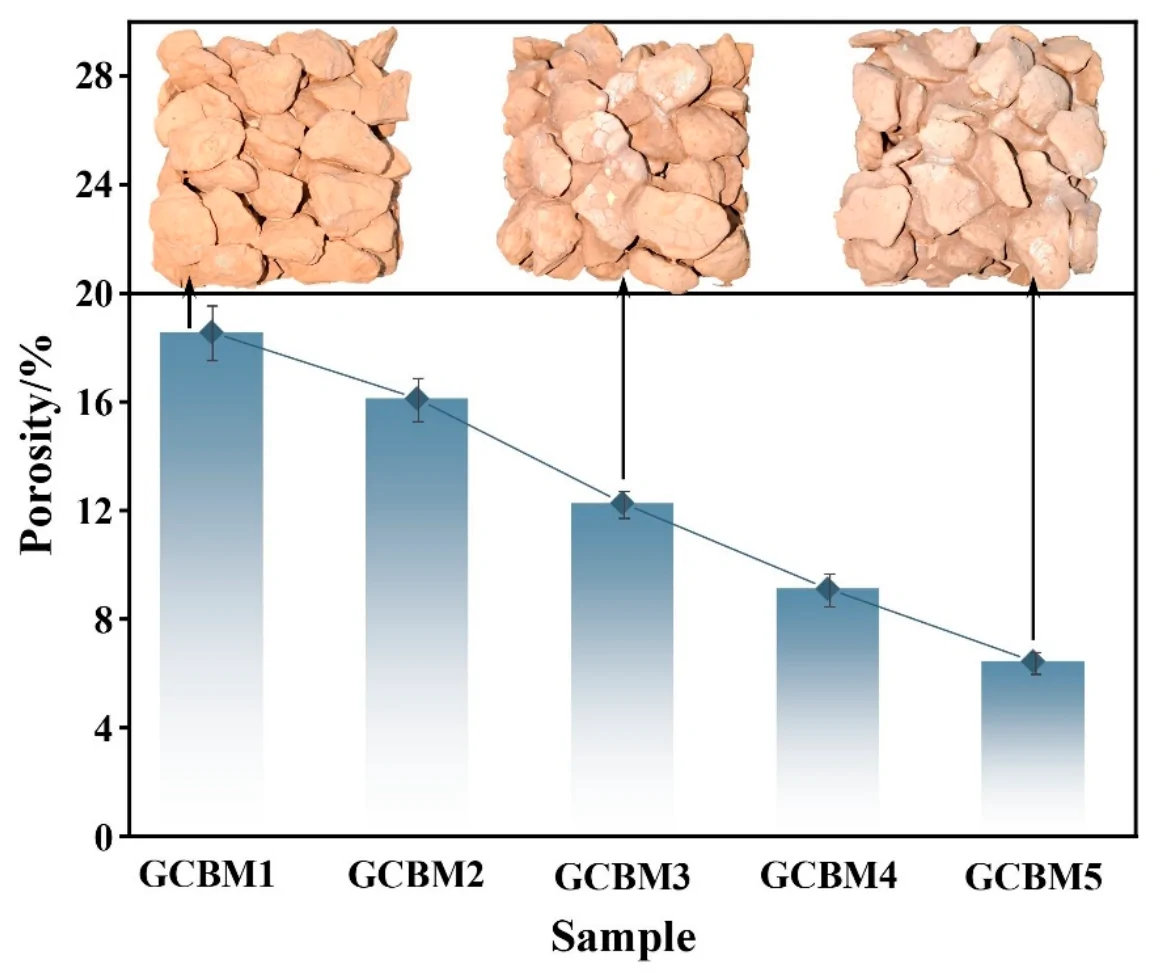
Influence of the FCM proportion on the porosity of GCBM specimens.

Figure 10 shows the effect of FCM proportion on the porosity of GCBM specimens. The porosity gradually reduces with increasing FCM proportion. To be specific, as the FCM proportion rises from 20% to 28%, the porosity of corresponding specimens successively decreases to 18.57%, 16.13%, 12.28%, 9.12%, and 6.45%. This intuitive trend in the porosity with changing FCM proportion is illustrated in Figure 10. The reduction in porosity is attributed to changes in the aggregate structure caused by the increasing FCM proportion: a higher FCM proportion increases the thickness of the effective coat on the aggregate surface. The thickening coat increases the thickness of the bonding layer between adjacent CG aggregates while also enlarging the effective contact area between aggregates. Under the combined effect, more pre-existing pores are effectively covered and filled by FCM with filling and bonding properties, thus finally inducing the reduction in the overall porosity of the specimens.

#### 3.2.2. Compressive and Flexural Strengths

**Figure 11:**
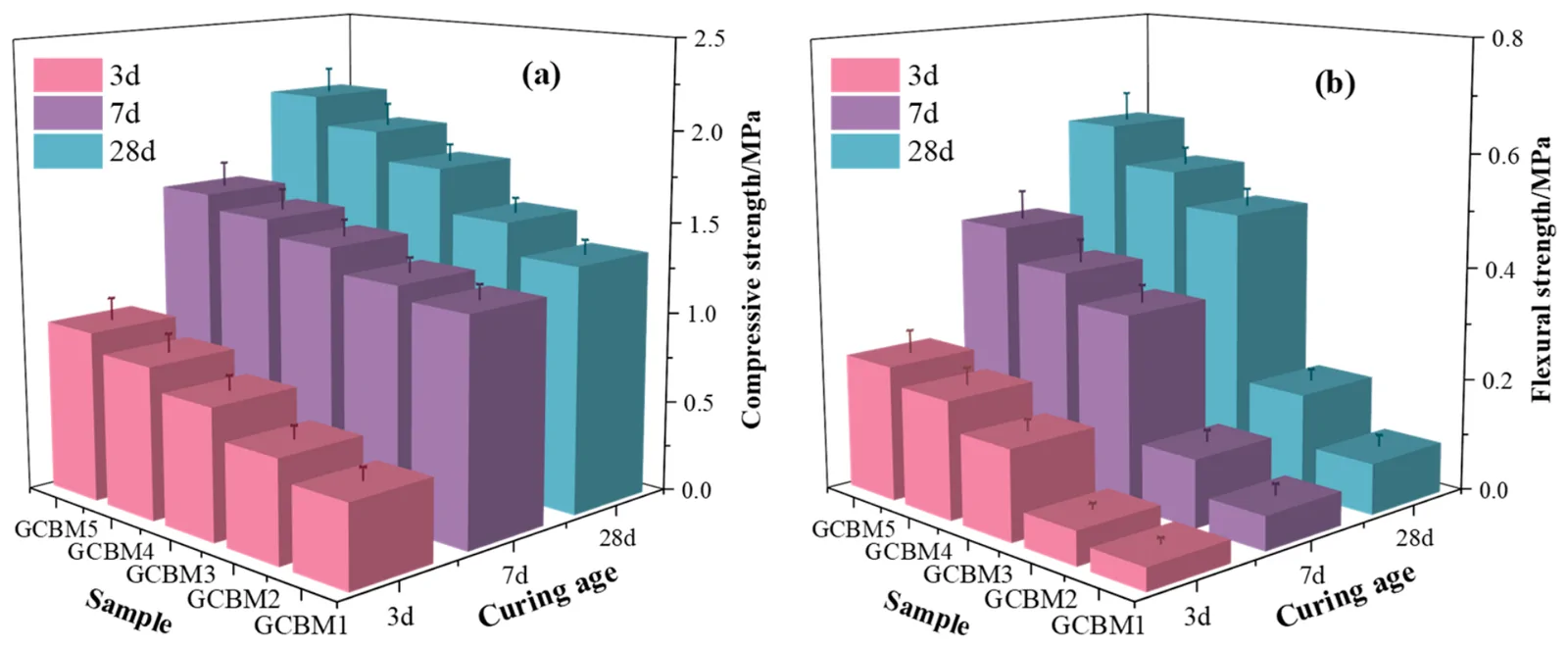
Effect of FCM proportion and curing age on the mechanical properties of GCBM: (**a**) compressive strength and (**b**) flexural strength.

The mechanical strength of GCBM is governed by the FCM proportion, which determines the thickness of the binding coat on CG aggregates. As shown in Figure 11, both compressive and flexural strengths at 3, 7, and 28 days exhibit a positive correlation with the FCM proportion and curing age. This trend is attributed to a thicker interfacial bonding layer and the concurrently reduced porosity (Figure 10). A critical enhancement occurs as the FCM proportion rises from 22% to 24%, most notably evidenced by a 172% surge in the 28-day flexural strength from 0.18 to 0.49 MPa. This is mainly because the flexural strength is more sensitive to the bonding performance of the aggregate–FCM interface. The bonding performance and continuity of the interface show qualitative improvement when the FCM proportion reaches the critical threshold (24%), thus effectively overcoming the weak links and significantly improving the flexural strength of the specimens [24].

The strength of GCBM specimens exhibits a consistent increase with prolonged curing, a trend that holds across all FCM proportions (Figure 11). This is primarily due to the continuous precipitation of hydration products like calcium silicate hydrate and ettringite, which condense on particle surfaces and form dense bonds, thereby enhancing interfacial strength [46]. However, the rate of strength gain diminishes by the 28-day mark. This deceleration is attributed to a decline in the system’s pH from OH^−^ consumption and the depletion of active silico-aluminous components, which collectively reduce the dissolution and reaction rates of aluminosilicates [47].

#### 3.2.3. Correlation Analysis of Properties

**Figure 12:**
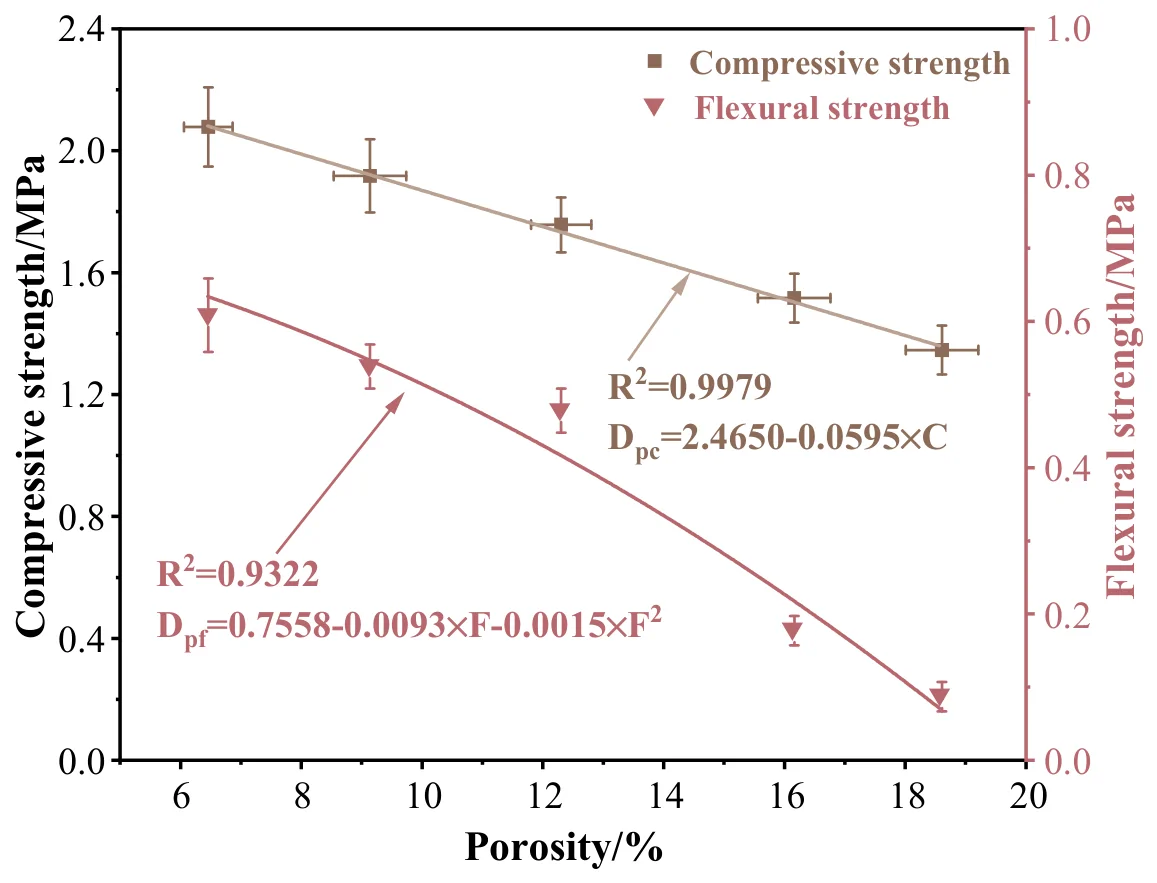
Correlations of the porosity with the compressive and flexural strengths.

Figure 12 displays the correlations of the mechanical properties of GCBM with the porosity. The mechanical properties are shown to be significantly negatively correlated with the porosity, with the coefficients of determination *R*^2^ separately being 0.9979 and 0.9322, indicative of a good fitting effect. The compressive strength of GCBM specimens shows a linear relationship with the porosity, meaning that it decreases at a quasi-constant rate with the increment of porosity; the flexural strength and porosity are related by a quadratic function, suggesting that the rate of deterioration of the flexural strength accelerates non-linearly with the increasing porosity. The specific equations are displayed in Figure 12. Compared with the compressive strength, the flexural strength is more sensitive to changes in the porosity, especially when the porosity exceeds 10%, whereupon the flexural strength declines abruptly. This matches the effect of the bonding threshold of interfaces described in Section 3.2.2.

### 3.3. The ITZ Analysis of GCBM

GCBM constitutes a three-phase composite of CG aggregates, an FCM matrix, and ITZ, forming a point-connected, honeycomb-like structure (Figure 10). Consequently, the ITZ microstructure, which is governed by the FCM hydration process, dictates the macroscopic mechanical performance of the material [48,49]. To this end, XRD analysis was employed to track the compositional evolution of FCM, offering insights into the overall properties. Complementary BSE-EDS characterization revealed the ITZ’s micro-morphology and elemental distribution, elucidating the structure–property relationships in GCBM.

**Figure 13:**
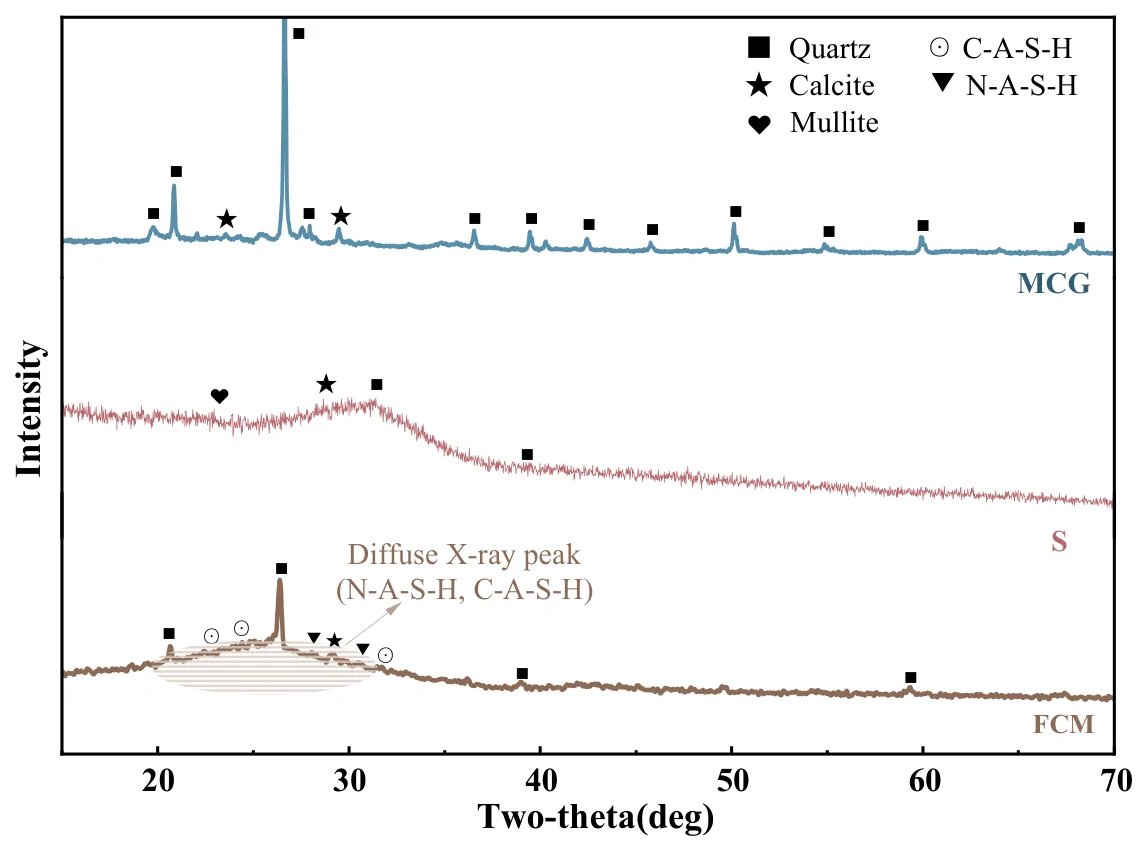
XRD patterns of MCG, S, and FCM.

Figure 13 illustrates the XRD patterns of MCG, S, and FCM. The main mineral components in MCG include quartz and kaolinite, which offer active SiO_2_ and Al_2_O_3_, while S is mainly composed of calcite and monticellite, which provide calcium sources for hydration reactions. After alkali activation, the diffraction peaks of quartz and mullite decrease in amplitude, while FCM cured for 28 d exhibits a wide diffuse peak at 20° ≤ 2*θ* ≤ 30°. This indicates the polyreaction of the alkali activator with active components in MCG and S, changes in the mineral component, and production of gel products dominated by C-A-S-H and N-A-S-H. These products play a key role in the development of strength in GCBM [50].

**Figure 14:**
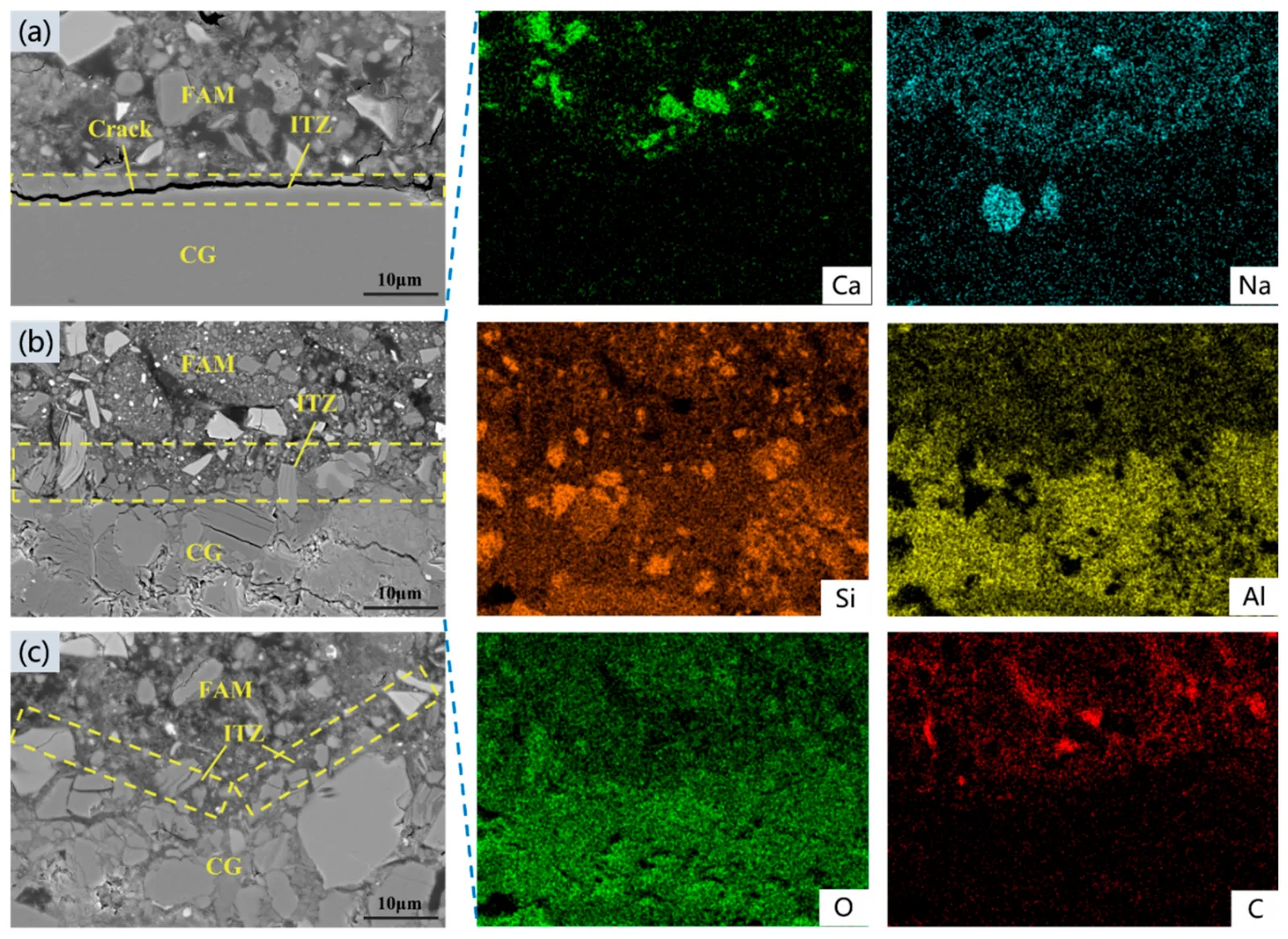
BSE images of (**a**) GCBM1, (**b**) GCBM3, and (**c**) GCBM5, and the EDS images of (**b**) GCBM3.

Increasing the FCM proportion from 20% to 28% leads to a markedly denser ITZ structure and stronger interfacial bonding, as evidenced by the BSE-EDS results in Figure 14. To be specific, the ITZ shows obvious fractures at an FCM proportion of 20% (GCBM1), suggesting poor interfacial bonding and thus a low interfacial strength. This is mainly because under a low FCM proportion, only limited amounts of gels are produced by hydration, which can thus only poorly coat the CG aggregates, so that fractures are likely to be initiated at the interface. Once the FCM proportion is increased to 24% (GCBM3) and 28% (GCBM5), the ITZ does not have obvious defects and is in a favorable interfacial bonding state. This is because the increased FCM, on the one hand, more fully fills those voids around CG aggregates and, on the other hand, forms a thicker, more complete gel coat, which significantly improves the compactness and integrity of the ITZ. This microstructural change aligns with the improvement in macroscopic mechanical properties described in Section 3.1.2.

Further EDS analysis (Figure 14b) demonstrates that the ITZ is rich in Ca, Na, Si, Al, and O. Combined with XRD analysis results, it is deduced that these elements not only come from hydration products (such as C-A-S-H and N-A-S-H gels) of FCM itself but also are generated by the interfacial reaction of FCM with active SiO_2_ and Al_2_O_3_ on the CG aggregate surface. OH^-^ provided by the FCM hydration system promotes the dissolution of aluminosilicate on the aggregate surface, which then reacts with FCM to form secondary reaction products dominated by C-A-S-H and N-(C)-A-S-H in the ITZ [51]. A large quantity of these gel phases is formed at the interface, realizing mutual diffusion and chemical binding of FCM matrix and CG aggregates, thus significantly strengthening the interfacial bond and the overall mechanical properties of GCBM specimens.

## 4. Discussion

**Figure 15:**
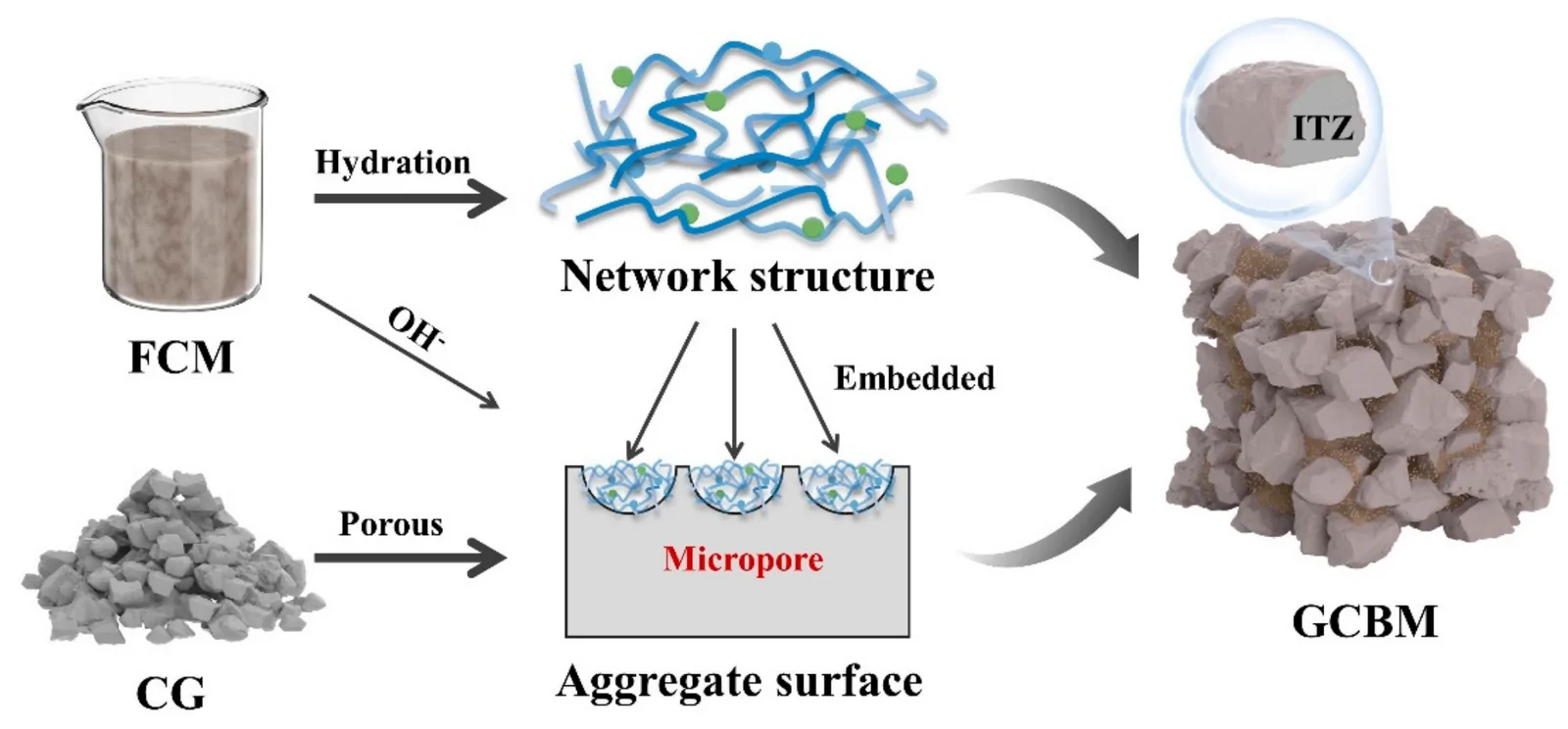
Interfacial bonding mechanism of GCBM.

In GCBM, which features an aggregate–matrix–aggregate sandwich structure, the Interfacial Transition Zone (ITZ) plays a pivotal role in stress transfer. The integrity of this zone governs the aggregate–matrix binding strength and, consequently, the macroscopic strength of the composite. A schematic summary of the resulting interfacial bonding mechanism, derived from ITZ analysis, is presented in Figure 15.

Aggregates have rough surfaces and contain widely distributed porous structures. When the FCM slurry is blended with CG, the slurry is likely to infiltrate the porous structures to link to CG aggregates more tightly and form a compact shell structure, which can seal the CG, significantly reduce the porosity of the ITZ, and form tighter microstructures. Meanwhile, the gels produced by the hydration of FCM can intertwine to form a network structure in the pores, which further strengthens the interfacial bonding, as illustrated in Figure 14c. Moreover, analysis of components of CG shows that CG contains high proportions of silicon oxide and aluminum oxide (Table 1). The OH^−^ environment provided by the FCM hydration system can promote dissolution of aluminosilicate on CG surfaces, which undergoes secondary reactions with FCM and produces reaction products dominated by C-A-S-H and N-(C)-A-S-H in the ITZ. These products do not impair the original structure of CG but effectively fill in micro-pores and micro-cracks instead, leading to an interfacial bonding mechanism of network structure between the aggregate and matrix, significantly enhancing the connectivity integrity and interfacial binding strength of the two. This mechanism also explains why GCBM prepared under a high FCM proportion shows a better interface density and overall properties.

## 5. Conclusions

The GCBM was developed with CG of between 15 and 20 mm as aggregates, which were cemented by FCM. This study investigated the effect of alkali equivalent on the workability and mechanical properties of FCM and examined the impact of FCM proportion on the porosity and mechanical performance of GCBM. A strength–porosity correlation model was established. The microstructural characteristics of the ITZ between the FCM matrix and CG aggregate were revealed from the microscopic perspective, and the mechanism of action of the interfacial effect on the macroscopic property of GCBM was expounded. The following main conclusions were obtained:

(1) When the alkali equivalent is 6%, FCM shows the optimal comprehensive properties. Under these conditions, the yield stress is 70.03 Pa, plastic viscosity is 1.15 Pa·s, initial and final setting times are 15 min and 24 min, and 28 d compressive strength reaches 3.31 MPa.
(2) The FCM proportion significantly influences the pore structure and mechanical properties of GCBM. As the FCM proportion is increased from 20% to 28%, the porosity of GCBM decreases from 18.57% to 6.45%, while the compressive and flexural strengths both increase significantly; the interface bonding changes abruptly when the FCM proportion reaches 24%, at which the 28 d flexural strength is 172% higher than that under the FCM proportion of 22%, suggesting the presence of a critical proportion threshold.
(3) The compressive strength of GCBM is linearly negatively correlated with the porosity (*R*^2^ = 0.9979), while the flexural strength follows a quadratic relationship with the porosity (*R*^2^ = 0.9322). The flexural strength is more sensitive to changes in the porosity, particularly when the porosity exceeds 10%, whereupon the flexural strength decreases abruptly.
(4) Microscopic analysis reveals that increasing the FCM proportion can optimize the ITZ structure. FCM fully fills the voids around CG aggregates and forms a thicker, more complete gel coat, which strengthens binding at the aggregate–matrix interface. Meanwhile, the secondary hydration reaction in the ITZ also plays a key role in improving the macroscopic mechanical properties of GCBM.

## Figures and Tables

**Table 1:** Chemical composition of CG, MCG and S, wt%.

Component	SiO_2_	Al_2_O_3_	Fe_2_O_3_	CaO	K_2_O	MgO	Na_2_O	TiO_2_	SO_3_	LOI
CG	57.37	24.61	7.08	3.06	2.82	1.06	0.59	1.12	1.08	6.35
MCG	57.62	26.33	6.04	4.19	2.69	1.07	0.53	1.07	0.66	0.17
S	30.48	17.03	0.58	33.47	0.47	14.44	0.37	0.79	2.37	1.21

**Table 2:** Mix proportions of GCBM specimens.

Sample	Particle Size of CG/mm	FCM Proportions/%	Water–Binder Ratio
GCBM1	15–20	*20%*	0.5
GCBM2		*22%*	
GCBM3		*24%*	
GCBM4		*26%*	
GCBM5		*28%*	

**Table 3:** Rheological parameters of FCM under different alkali equivalents.

Sample	Rheological Model	Fitting Results	*τ*_0_/Pa	*η*/Pa·s	*n*	*R* ^2^
FCM0%	H-B	*τ* = 61.75 + 1.05*γ*^0.86^	61.75	1.05	0.86	0.9996
FCM1%		*τ* = 35.51 + 0.44*γ*^0.91^	35.51	0.44	0.91	0.999
FCM2%		*τ* = 35.51 + 0.14*γ*^0.94^	35.51	0.14	0.94	0.9989
FCM4%		*τ* = 32.75 + 0.03*γ*^0.98^	32.75	0.03	0.98	0.9995
FCM6%		*τ* = 70.03 + 1.15*γ*^0.95^	70.03	1.15	0.95	0.9992
FCM8%		*τ* = 105.07 + 2.92*γ*^0.80^	105.07	2.92	0.80	0.9993

## Data Availability

The original contributions presented in this study are included in the article. Further inquiries can be directed to the corresponding author.
